# Seroprevalence and genotype of *Toxoplasma gondii* in pigs, dogs and cats from Guizhou province, Southwest China

**DOI:** 10.1186/s13071-015-0809-2

**Published:** 2015-04-10

**Authors:** Yong-Nian Li, XinWen Nie, Qun-Yi Peng, Xiao-Qiong Mu, Ming Zhang, Meng-Yuan Tian, Shao-ju Min

**Affiliations:** Department of Immunology, Guiyang Medical College, Guiyang, 550001 China; Department of Laboratory, Guiyang Medical College, Guiyang, 550001 China; Department of Clinical Laboratory, The Fourth Hospital of Guiyang, Guiyang, 550004 China; Department of Clinical Laboratory, Baiyun Hospital Affiliated to Guiyang Medical College, Guiyang, 264000 China

**Keywords:** Toxoplasma gondii, Animal, Seroprevalence, Genotype, Guizhou province

## Abstract

**Background:**

*Toxoplasma gondii* is an obligate, intracellular protozoan that infects almost all warm-blooded animals, including humans, domesticated and wild animals. Recent studies of *Toxoplasma gondii* isolates from animals in different regions of China have shown a limited genetic diversity with the dominance of the ToxoDB PCR-RFLP genotype #9 named as “Chinese 1”. However, there is not much published information regarding its prevalence in domestic animals from Guizhou province, a subtropical region in Southwest China. The objectives of this study were to determine seroprevalence and genetic diversity of *T .gondii* in pigs, dogs and cats in Guizhou province, Southwest China.

**Findings:**

The anti-*T. gondii* IgG were detected in 70.0%(49/70) pigs, 20.56%(22/107) dogs and 63.16(12/19) cats. The anti-*T. gondii* IgM were found in 0.93%(1/107) dogs, 21.53%(4/19) cats, but not in pigs. In addition, the toxoplasma circulating antigen (CAG) were detected in 16.9%18/70)pigs, 13.1% (14/107) dogs and 10.5%(2/19) cats. The *T. gondii* DNA were detected in 31.5%(22/70) pigs, 3.7%(4/107) dogs and 52.63%(10/19) cats. Five *T. gondii* isolates were obtained(3 from pigs and 2 from cats). The genotype of these five isolates belonged to the predominant genotype “Chinese 1”.

**Conclusions:**

The high prevalence of *T. gondii* infection in pigs,cats and dogs indicated that the *T. gondii* infection is common in Guizhou province. Additionally, the *T. gondii* genotype “Chinese 1” was dominant in Southwest China.

**Electronic supplementary material:**

The online version of this article (doi:10.1186/s13071-015-0809-2) contains supplementary material, which is available to authorized users.

## Findings

*Toxoplasma gondii* is an obligate, intracellular protozoan that infects almost all warm-blooded animals, including humans, domesticated and wild animals [1,2]. These animals can serve as intermediate hosts of the parasite, harbouring tissue cysts, while cats and other felidae are the definitive hosts, shedding oocysts into the environment. Humans acquire *T. gondii* through the consumption of undercooked meat containing tissue cysts or through the ingestion of sporulated oocysts that can lead to life threatening disease in the foetus and immunocompromised/immunosuppressed patients e.g. transplant recipients. In general, *T. gondii* is an opportunistic pathogen and establishes long-lasting chronic infection. However, *T. gondii* infection can cause high mortality in immunocompromised patients with HIV/AIDS.

The pathogenicity of T. gondii is related to parasite genotypes and susceptibility of host species [3]. Based on early molecular genotyping studies, *T. gondii* isolates in North America and Europe have been classified into three genetic types (I, II, III). TypeIisolates are lethal to mice, and typeIIand III are usually less virulent for mice [4]. High genetic diversity of T. gondii exists in Central and South America where a large number of genotypes were identified by RFLP typing [5]. To date, the three archetypical (type I, II and III) and several atypical types have been identified in China, of which the “Chinese 1” seems to be a predominant type [6].

Guizhou province is located in Yunnan-Guizhou plateau in Southwest China. Previous studies showed high seroprevalence of T. gondii in pigs and human in Guizhou province [7,8]. However the data on *T. gondii* is still limited. Especially as, there is no epidemiological or genotype information on *T. gondii* in animals here. Thus, the aim of the present study was to analyse the prevalence and genetic characteristics of *T. gondii* in domestic pigs, pet dogs and stray cats in Guizhou province, southwestern China.

In the present study, animal samples (blood, heart and brain tissues) were obtained from 70 pigs, 107 dogs and 19 cats from November, 2011 to December, 2012. The blood and heart tissues of pigs were collected from Guiyang Jiawang slaughterhouse. The dog blood samples were obtained from Guiyang Dear pet clinic. The cat’s blood and brain tissues were collected from stray cats, which were captured from some neighbourhoods in Guizhou province and the cats were euthanized. The anti-*T. gondii* IgG and IgM antibodies and the toxoplasma circulating antigens (CAG) were assayed by Toxoplasma ELISA Test Kits (Zhuhai Haitai Life Technology Company, China). DNA was extracted from heart or brain tissue (5 g), or blood (1 ml) sample for PCR detection of the 529 bp repetitive DNA element of T. gondi. DNA extraction was performed using DNA extraction reagent kits (Beijing Ding changsheng biotechnology company, China). The tissue sample homogenates (5 g/10 ml) from seropositive animals were bioassayed in mice for isolation of the *T. gondii* strain, following the previously described protocol [6,9]. Mouse peritoneal exudates were collected and examined for viable *T. gondii*. Tissue cysts were microscopically examined as a squash preparation as described previously [10]. *T. gondii* tachyzoites collected from intraperitoneal fluid were cryopreserved in liquid nitrogen for long term storage. Genotyping of *T. gondii* isolates was performed using multilocus PCR-RFLP with 10 genetic markers as previously described [4]: SAG1, SAG2, SAG3, BTUB, GRA6, c22-8, c29-2, L358, PK1 and Apico. Reference strains of *T. gondii* were also used in genotyping, including type I (GT1), type II (PTG), type III (CTG) and other strains (MAS, TgCgCa1, TgCatBr5, TgWtdsc40, TgToucan(TgrRsCr1), and TgCatBr64) were kindly provided by Dr. Chunlei Su at the University of Tennessee, Knoxville USA. In addition, UPRT-1 intron sequence of *T. gondii* was amplified through nested-PCR. The DNA sequencing was generated by SinoGenoMax company (Beijing, China). The PCR products were digested with `appropriate restriction endonucleases. The restriction fragments were run by electrophoresis. And the typing data were analyzed using ToxoDB (www.toxodb.org) database and compared with the reference strain profiles.

**Figure 1:**
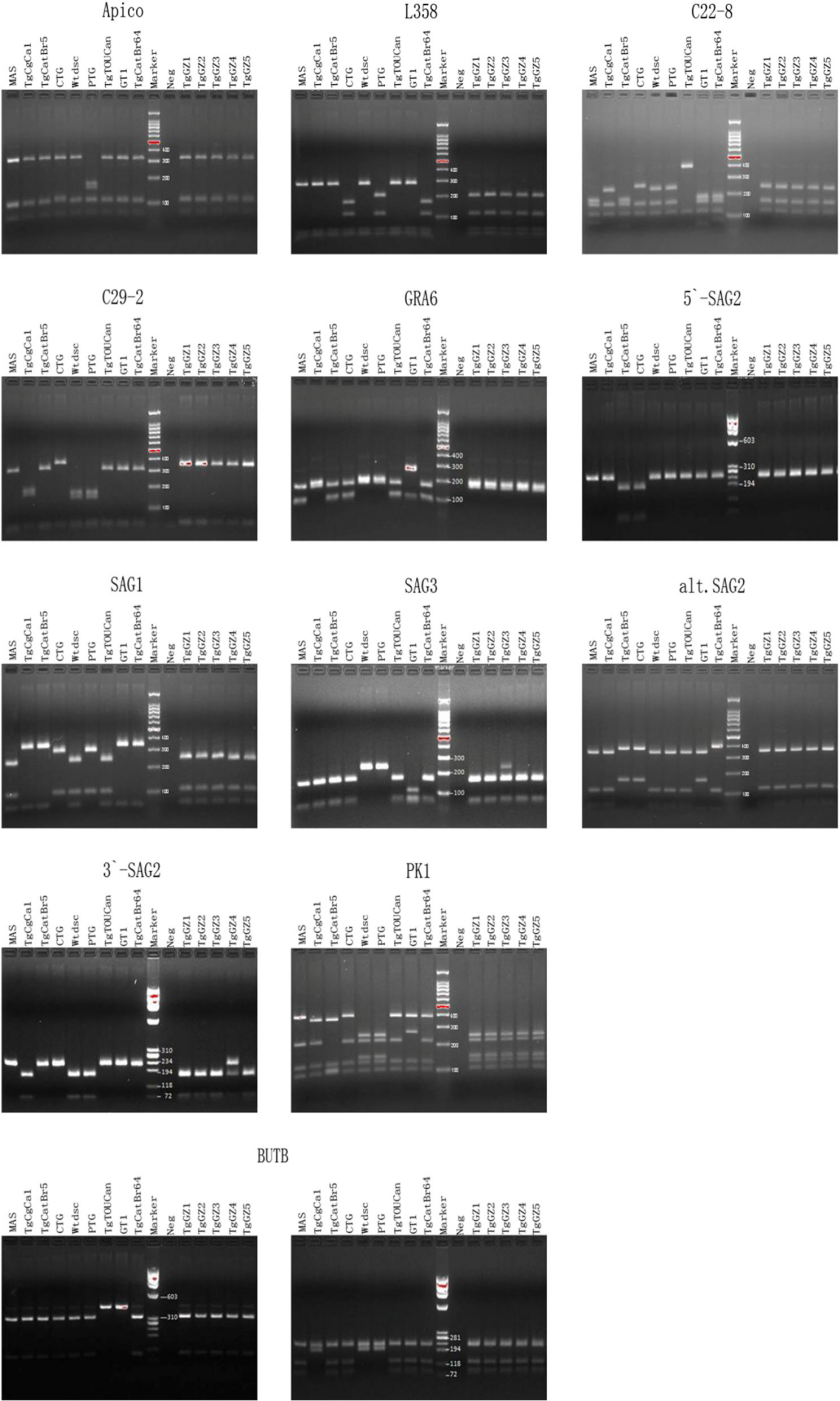
**Multiplex multilocus nested PCR-RFLP (Mn-PCR-RFLP) analysis of Toxoplasma gondii isolates and reference strains.** (reference strains are GT1, PTG, CTG, MAS, TgWtdSc40, TgCgCa1, TgToucan, TgCatBr5, TgCatBr64. isolates are TgGZ1- TgGZ5).

In this study, Toxoplasma specific IgG, IgM, CAG and 529 bp DNA fragments were tested in 196 animals including 70 pigs, 19 cats and 97 pet dogs. The anti-*T. gondii* IgG were detected in 70.0% (49/70) pigs, 20. 6% (22/107) dogs and 63.2% (12/19) in cats. The anti-*T. gondii* IgM were not found in pigs, but were found in 0.9% (1/107) of dogs and 21.5%(4/19) of cats tested. In addition, the toxoplasma circulating antigen (CAG) positive rate was 16.9%(18/70) in pigs, 13.1% (14/107) in dogs and 10.5%(2/19) in cats. The *T. gondii* DNA were detected in 31.5%(22/70) pigs, 3.7% (4/107) dogs and 52.6% (10/19) cats (Table 1). Furthermore, five viable *T. gondii* isolates were obtained (3 from pigs and 2 from cats). These isolates displayed the identical genotype, which belongs to the Chinese 1 type. The UPRT-1 sequences from these isolates are identical and confirmed as the Chinese 1 genotype, a dominant type in China [6,8] (Figure 1 and Table 2).

**Table 1:** **Serological test and 529 bp detection of** ***T. gondii*** **in pigs, cats and dogs**

**Animals**	**Quantities**	**IgG positive**		**IgM positive**		**CAG positive**		**529bpPCR positive**	
		**Samples**	**%**	**Samples**	**%**	**Samples**	**%**	**Samples**	**%**
Pigs	70	49	70	0	0	18	16.88	22	31.51
Straying cats	19	12	63.16	4	21.05	2	10.53	10	52.63
Pet dogs	97	19	19.59	1	1.03	14	14.43	4	4.12
Total	186	80	43.01	5	2.69	34	18.28	36	17.2

**Table 2:** **Genotype of** ***T. gondii*** **reference strains and isolates in Guizhou province**

**Reference strains and isolates**	**Markers**											
	**SAG1**	**(5’ + 3’)SAG2**	**Alt.SAG2**	**SAG3**	**BTUB**	**GRA6**	**C22-8**	**C29-2**	**L358**	**PK1**	**Apico**	**Comments**
GT1,RH88(typeI)	I	I	I	I	I	I	I	I	I	I	I	Reference
PTG(type II)	IIorIII	II	II	II	II	II	II	II	II	II	II	Reference
CTG(type III)	IIorIII	III	III	III	III	III	III	III	III	III	III	Reference
MAS	μ-1	I	II	III	III	III	μ-1	I	I	III	I	Reference
TgWtdSc40	μ-1	II	II	II	II	II	II	II	I	II	I	Reference
TgCgCa1	I	II	II	III	II	II	II	μ-1	I	μ-2	I	Reference
TgRsCr1	μ-1	I	II	III	I	III	μ-2	I	I	III	I	Reference
TgCatBr5	I	III	III	III	III	III	I	I	I	μ-1	I	Reference
TgCatBr64	I	I	μ-1	III	III	III	μ-1	I	III	III	I	Reference
TGGZ1(pig)	μ-1	II	II	III	III	II	II	III	II	II	I	This study
TGGZ2(pig)	μ-1	II	II	III	III	II	II	III	II	II	I	This study
TGGZ3(pig)	μ-1	II	II	III	III	II	II	III	II	II	I	This study
TGGZ4(cat)	μ-1	II	II	III	III	II	II	III	II	II	I	This study
TGGZ5(cat)	μ-1	II	II	III	III	II	II	III	II	II	I	This study

(reference strains are GT1, PTG, CTG, MAS, TgWtdSc40, TgCgCa1, TgToucan, TgCatBr5, TgCatBr64. isolates are TgGZ1- TgGZ5).

The present results showed high prevalence of T. gondii (70.0%) in pigs. It is in agreement with previous reported prevalence of 65.8% in pigs in Guizhou province [8], and 60.4% in Chongqing [11]. This high prevalence level can be explained by poor-managed facilities in this area. It was shown that if rodents and cats were controlled, as carried out on well-managed intensive farms, T. gondii prevalence would drop drastically, in a similar way to that observed in the USA and other developed countries [12,13]. The high prevalence of T. gondii in pigs from different farms, that were often infested with rats and cats, seems to correlate well with the high prevalence of T. gondii in stray cats. Here, we show that 12/19 (63.2%) of stray cats were infected with T. gondii. The prevalence of T. gondii infection in stray cats was 63.2% in Guiyang, 57.8% in Beijing [14], 45.3% in Lanzhou [15] and 11.7% in Shanghai [16]. Whereas it was reported that 19.5% of pet dogs in Guiyang, 13.2% in Beijing [17], 11.1% in Lanzhou [18] and 2.6% in Shanghai were infected [19]. In general, higher prevalence in cats was accompanied by higher prevalence in humans, dogs, pigs and other susceptible animals, therefore increasing the chances of environmental contamination by millions of oocysts shed by infected cats, and higher risk of ingestion of meats containing tissue cysts from infected animals [20]. Therefore, controlling the T. gondii infection and contamination emission of cats is important.

There is scarce information concerning the isolation and genotyping of *T. gondii* in Guizhou province. In the present investigation, we obtained five viable *T. gondii* isolates (3 from pigs and 2 from cats) by bioassay in mouse. These isolates showed low virulence in mice(the data will reported in another paper). Furthermore, these isolates have identical genotype and belongs to “Chinese 1”. Previous reports of genetic typing of *T. gondii* isolates from cats in China revealed that 15 (total 28, 88.23%) isolates are “Chinese 1” [13]. This genotype has also been found in Guangdong province, and Hunan, and Hubei province in China. Especially in Guangdong province, 26 (total 28, 92.86%) isolates were “Chinese 1” indicating it was the dominant genotype in that region [21]. The recent literature on genotypes revealed that 15/23(65.2%) of T. gondii isolates from Anhui, and Hubei, and Guizhou province were the “Chinese 1”. At the same time, typesI,II,III and other atypical types were also found in these areas [6,22]. Our results confirm that the “Chinese 1” is a dominant isolate that is wide spread in China.

### Ethics statement

All experimental animals were treated in strict accordance to the guidelines for the Laboratory Animal Use and Care from Chinese CDC and the Rules for Medical laboratory Animals (1998) from Ministry of Health, China. The protocols were approved by the Institutional Review Board (IRB) of the Institute of Biomedicine at Guiyang Medical College. All efforts were made to minimize animal suffering during the course of these studies.
